# Self-Referential Information Alleviates Retrieval Inhibition of Directed Forgetting Effects—An ERP Evidence of Source Memory

**DOI:** 10.3389/fnbeh.2017.00187

**Published:** 2017-10-10

**Authors:** Xinrui Mao, Yujuan Wang, Yanhong Wu, Chunyan Guo

**Affiliations:** ^1^School of Psychological and Cognitive Sciences, Peking University, Beijing, China; ^2^Beijing Key Laboratory of Learning and Cognition, Department of Psychology, Capital Normal University, Beijing, China; ^3^Beijing Advanced Innovation Center for Imaging Technology, Capital Normal University, Beijing, China

**Keywords:** directed forgetting, retrieval, ERP, self-referential information, source memory

## Abstract

Directed forgetting (DF) assists in preventing outdated information from interfering with cognitive processing. Previous studies pointed that self-referential items alleviated DF effects due to the elaboration of encoding processes. However, the retrieval mechanism of this phenomenon remains unknown. Based on the dual-process framework of recognition, the retrieval of self-referential information was involved in familiarity and recollection. Using source memory tasks combined with event-related potential (ERP) recording, our research investigated the retrieval processes of alleviative DF effects elicited by self-referential information. The FN400 (frontal negativity at 400 ms) is a frontal potential at 300–500 ms related to familiarity and the late positive complex (LPC) is a later parietal potential at 500–800 ms related to recollection. The FN400 effects of source memory suggested that familiarity processes were promoted by self-referential effects without the modulation of to-be-forgotten (TBF) instruction. The ERP results of DF effects were involved with LPCs of source memory, which indexed retrieval processing of recollection. The other-referential source memory of TBF instruction caused the absence of LPC effects, while the self-referential source memory of TBF instruction still elicited the significant LPC effects. Therefore, our neural findings suggested that self-referential processing improved both familiarity and recollection. Furthermore, the self-referential processing advantage which was caused by the autobiographical retrieval alleviated retrieval inhibition of DF, supporting that the self-referential source memory alleviated DF effects.

## Introduction

Forgetting can be considered an adaptive strategy in preventing traumatic or outdated information from interfering with current cognitive processing (Bjork, 1989). However, self-referential details are often hard to forget in real life (i.e., the autobiographical experience of my ex-girlfriend). Some researchers have focused on the neurocognitive mechanism of memory encoding processing, demonstrating that self-referential items are harder to forget than other-referential item (Yang et al., 2013). However, the neurocognitive mechanism of memory retrieval processing which elicits this phenomenon is not clear.

Directed forgetting (DF) effects are demonstrated by poorer recall and recognition of TBF items (“to-be-forgotten”) than TBR items (“to-be-remembered”; Van Hooff et al., 2009). To explore intentional forgetting, a typical paradigm of “DF” is used in the experiment (MacLeod, 1998). This procedure provides two explicit cues to index TBR items and TBF items during the study phase. These two cues can be presented either after each item (item-method) or following the entire list of items (list-method). For the item methods, these effects are generally accounted for both selective rehearsal hypothesis and retrieval inhibition hypothesis. The selective rehearsal hypothesis suggests that DF stems entirely from the diminished elaboration or rehearsal of TBF rather than TBR words (Woodward et al., 1973; Basden and Gargano, 1993), and the retrieval inhibition hypothesis is that DF suppresses the retrieval of these items (Levy and Anderson, 2008; Mecklinger et al., 2009).

It is generally acknowledged that DF effects are alleviated by self-relevant information due to self-referential effects. The self-referential effect reflects self-relevant events that are remembered better than events related to other events (van den Bos et al., 2010; Cunningham et al., 2011; Klein, 2012). This retrieval advantage of the self-referential effect can be explained by the observation that self-referential processing tasks make the encoding of information more effective, involving elaboration and organization strategies, and the subsequent memory retrieval improves as a result (Rogers et al., 1977; Symons and Johnson, 1997). Because the diminishing elaborative encoding of TBF instruction can be improved by self-referential processing, self-referential information can alleviate DF effects.

In contrast to the encoding account, it is not clear that the mechanism of memory retrieval processing elicits this phenomenon. Based on dual-process framework of recognition, recognition retrieval included two types of process: familiarity and recollection. Familiarity is a fast and automatic retrieval process that does not require details and recollection is a slower process that supports the conscious retrieval of specific episodic details (Yonelinas, 2002). When retrieval of self-referential information relies on familiarity without any details, recognition judgments are made via access to semantic self-knowledge (Klein, 2012). For example, when people made judgments for self-trait and the self-trait was paired with its matching semantic self-knowledge, they benefited from fast retrieval without any contextual details. By contrast, when the retrieval of self-referential information relies on recollection, the “autobiographical retrieval” of episodic details is more likely to play an important role in the judgment of recognition. The autobiographical retrieval consists of specific items of personal information that are closely related to unique autobiographical events which refer to the individual in relation to a specific episodic context (Matuszewski et al., 2009). Particularly, the self-referential effects with nouns are typically obtained with autobiographical retrieval promoting to recollect more episodic details of self-referential information (Klein, 2012). According to retrieval inhibition hypothesis of DF, sub-processes (familiarity vs. recollection) of retrieval inhibition which could be overcome by self-referential effects need to be investigated.

In order to explore the retrieval mechanism, source memory tasks and event-related potential (ERP) measurements were used in the current study. Based upon memory distinction between central items of an event (i.e., item memory) and contextual details of the event (i.e., source memory), source memory tasks were sensitive to examine the recollection of specific contextual details (Sahakyan and Delaney, 2005; Racsmány and Conway, 2006; Racsmány et al., 2008). For scalp electrophysiological recording, three important ERP components were involved in recognition retrieval of source memory: the FN400 (a positive shift or reduction in negativity in frontal regions at 300–500 ms) that indexed familiarity, the late positive complex (LPC; a positive component over posterior regions at 500–800 ms) that indexed recollection, and the late right frontal effect (an amplitude maximum over right frontal scalp at 800–1200 ms) that indexed post-retrieval monitoring (Curran, 2000; Diana et al., 2007; Rugg and Curran, 2007). The right frontal effect indicated the episodic reorganization and source monitoring of the retrieval (Hayama et al., 2008; Cruse and Wilding, 2009).

Therefore, we combined a DF task (item method) with a source memory task so that we could investigate neurocognitive mechanism of the alleviative forgetting of self-referential information. Specifically, the retrieval processes (familiarity vs. recollection) of DF effects which could be modulated by self-referential processing should be demonstrated. Besides being associated with self-referential effects, recollection might possibly be involved in the DF effects which were reduced by self-referential processing, because previous ERP and behavioral evidences supported retrieval inhibition of impaired recollection of episodic details (“episodic inhibition”). In behavioral researches, DF effects were only present in responses accompanied by recollected experience in a recognition test (Racsmány et al., 2008). The retrieval inhibition of TBF items was observed in the absence of the old/new effect at parietal sites which reflected recollection processing (Ullsperger et al., 2000; Van Hooff et al., 2009; Xiao et al., 2012). So, the following hypotheses were tested. Given that the self-referential advantages of retrieval rely upon both semantic self-knowledge and autobiographical retrieval (Uncapher and Rugg, 2009; Turk et al., 2013), we investigated the possibility that self-referential information would elicit enhanced behavioral accuracies and increased ERP amplitudes of FN400 (indexing familiarity) and LPC (indexing recollection) effects. As DF effects were caused by retrieval inhibition of episodic details, we also assumed that the recollection processes would be associated with alleviative DF which are caused by self-referential processing.

## Materials and Methods

### Participants

Nineteen college students from Capital Normal University (Beijing, China) with normal or corrected-to-normal vision participated in this study. These subjects were right-handed, native Chinese speakers with no history of psychiatric or neurological disorders, head injury, or psychotropic drug use. Three participants were excluded (their artifact-free ERP trials were less than 18 in at least one condition, due to excessive eye movements or EEG artifacts), leaving a final sample of 16 participants (mean age, 22.2 years; 8 men). Each subject signed an informed consent form before experiment and received monetary compensation after experiment. This study was carried out in accordance with the recommendations of “Human Research Ethics Committee at Capital Normal University” with written informed consent from all subjects. All subjects gave written informed consent in accordance with the Declaration of Helsinki. The protocol was approved by the “Human Research Ethics Committee at Capital Normal University”. No additional considerations of the study in cases where vulnerable populations were involved.

### Materials

Two-character Chinese nouns (640 in total) were used as stimuli (mean total number of strokes: 16.51 (ranging from 5 to 35), mean word frequency: 14.79 (ranging from 2.3 to 99.7) occurrences per million (Liu et al., 1990)). Fourteen adult native Chinese speakers (an independent sample; 7 men) provided concreteness ratings of the nouns. The concreteness ratings (from 1/extremely abstract to 7/extremely concrete) confirmed that the set of nouns was concrete nouns (Mean = 6.12, ranging from 5 to 6.92). All nouns were separated into two equal sets (320 nouns for set A and 320 nouns for set B) that were alternatively used as “old” (studied) or “new” (unstudied) items at the test, which was counterbalanced across participants. The items of set A and B were randomly selected to have equivalent concreteness, number of strokes or word frequency.

### Experimental Procedure

**Figure 1:**
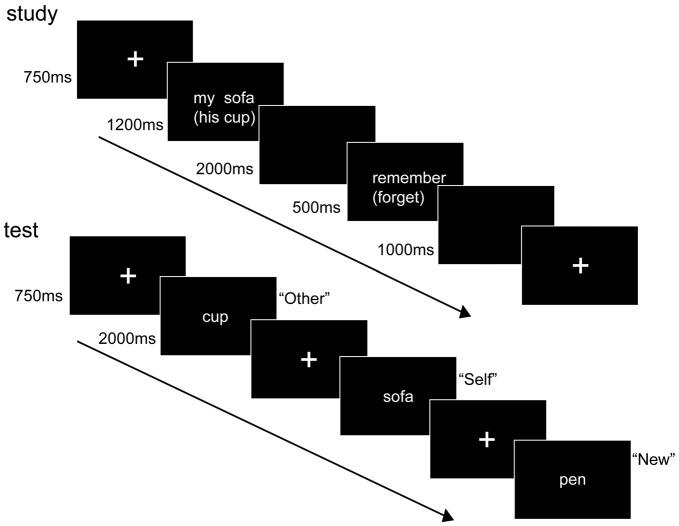
Experimental paradigm. In our experiment, participants completed a study phase immediately followed by a test phase. During the study phase, participants were explicitly instructed to follow the instruction to remember “to-be-remembered”, (TBR) both the noun and its source (personal pronoun) or to forget “to-be-forgotten”, (TBF) them. During the test phase, participants were asked to make a response of Self (S), Other (O) or New (N).

Participants were seated 70 cm from a Dell monitor in an electrically shielded room wherein they performed an extrinsic source memory task (Figure 1). After a short practice block, participants undertook the experiment, which included five study-test blocks (64 old and 64 new items in total), with each study phase followed by a test phase after a 2-min gap. The studied items were presented in two separate referential categories (“self” vs. “other”). In the “self” condition, the nouns were accompanied with the personal pronoun “my” (such as “my cup”, “my table” etc.); in the “other” condition, the nouns were presented together with the personal pronoun “his” (such as “his sofa”, “his pen” etc.). Participants were instructed to consider “my-noun” pairs as the things related to themselves (indicating self-reference) and to consider “his-noun” pairs as the things related to Li Keqiang (Chinese Prime Minister; indicating other-reference).

Each block consisted of a study and a test phase. During the study phase, each trial began with a fixation cross (750 ms) that was followed by a pronoun-noun pair (“my-noun” pair or “his-noun” pair, extending a 9° × 7° visual area), which was centrally presented for 1200 ms. After pronoun-noun pair, a blank screen, which lasted for 2000 ms, was inserted. Then, an instruction “remember” (indexing TBR) or “forget” (indexing TBF) was displayed for 500 ms. At the end of each trial, another blank screen was shown for 1000 ms. All stimuli were presented in white against a black background. The order of trials was pseudo-random with each type of instruction (TBR vs. TBF) appearing in no more than three consecutive trials. Additionally, stimulus types of reference (“my–noun” and “his–noun” pairs) were presented randomly in every session. Participants were explicitly instructed to follow the instruction to remember both the noun and its source (personal pronoun) or to forget them.

Two minutes after the study phase, the participants’ source memory was tested. In each block, the test stimuli consisted of 64 old items (two-character Chinese noun presented in the study phase) and 64 new items. All 128 nouns were pseudo-randomly intermixed. In each trial, after a fixation cross for 750 ms, a noun (single word without any pronoun, extending an 8° × 7° visual area) was presented for 2000 ms. During the presentation of the noun, the participants were asked to make a response of Self (S), Other (O) or New (N), thereby indicating whether the noun belonged to the self (“my-noun” pair) or other (“his-noun” pair) category or whether the noun was never presented. Three out of the four letter keys on a standard English keyboard (i.e., “D”, “F”, “J”, “K”) were chosen as response keys for the three possible responses (i.e., S, O, N). The S/O response keys were assigned to one hand, and the N keys to the other hand. The response hand assignment was counterbalanced across participants.

### Behavioral Analysis

Self-old items that required S responses and were assigned to correct sources were deemed to be “self-source-correct” (SSC) responses. Other-old items that required O responses and were assigned to correct sources were considered “other-source-correct” (OSC) responses. Finally, new items that required N (new) responses were considered “correct rejections” (CR). Source accuracy for the TBR instruction and TBF instruction was calculated as the ratio of the number of source correct items with the TBR and TBF instruction, respectively, over its corresponding number of hit items. Two-way repeated-measures analysis of variance (ANOVA; with Greenhouse-Geisser corrections) was performed on source accuracy, with independent variables of reference (self, other) and instruction type (“TBR”, “TBF”). Significant main and interaction effects were followed by Bonferroni-corrected *post hoc* pair-wise comparisons.

### EEG Recording and Analysis

The continuous electroencephalography recordings were measured during test phases. According to the extended international 10–20 system, 64 scalp sites of Ag/AgCl electrodes embedded in an elastic cap. An electrooculogram (EOG) was recorded with four additional channels which were used to monitor horizontal and vertical eye movements. EEG signals were referenced to the left mastoid during recording and re-referenced offline to the average of the left and right mastoid recordings. EEG/EOG signals (impedance <5 kΩ) were digital bandpass filtered from 0.05 Hz to 40 Hz, segmented around image onset (−200 to 1600 ms) and corrected to a 200 ms pre-stimulus baseline. Trials with EEG voltages that exceeded ±75 μV were excluded from analysis. EOG blink artifacts were corrected using a linear regression estimate (Gratton et al., 1983). Experiment presentation was executed using Presentation (Neurobehavioral Systems, Inc.). Data collection was performed using Neuroscan acquisition software, and statistical analysis was performed in SPSS 20.0.

Mean ERP amplitudes were extracted from three time windows (300–500 ms, 500–800 ms, 800–1200 ms after test item onset) to estimate the old–new effect, as indexed by the FN400, LPC and late right frontal effect. The time windows were selected based upon both visual inspection of the grand average ERP waveform and previous ERP literature on familiarity (FN400) and recollection (LPC; Rugg and Curran, 2007; Voss and Paller, 2008). Electrodes were selected around frontal, central and parietal sites (anterior site: F1, F2, F3, F4, Fz; central sites: C1, C2, C3, C4, Cz; posterior site: P1, P2, P3, P4, Pz). We computed ERP amplitudes of source correct trials (SC) to index source memory (Guo et al., 2006; Addante et al., 2012). The ERPs of source memory were classified on the basis of the subject’s behavior into one of five subsets: TBR_SSC (self-correct source of TBR instruction), TBR_OSC (other-correct source of TBR instruction), TBF_SSC (self-correct source of TBF instruction) TBF_OSC (other-correct source of TBF instruction *n*) and CR (correct rejection) trials. However, there were not enough TBR_SSI (*M* = 11.70, SD = 2.07; self-incorrect source of TBR instruction), TBR_OSI (*M* = 12.30, SD = 2.05; other-incorrect source of TBR instruction), TBF_SSI (*M* = 14.20, SD = 2.11; self-incorrect source of TBF instruction), and TBF_OSI (*M* = 14.35, SD = 2.12; other-incorrect source of TBF instruction) trials for the averaging procedure to result in a reliable ERP. In order to obtain grand-average ERP, the ERPs of one type from each subject and each recording site were averaged. Repeated-measures ANOVA included Greenhouse–Geisser corrections when necessary and Bonferroni-corrected *post hoc* pair-wise comparisons.

## Results

### Behavioral Results

A two-way repeated-measures ANOVA of reference and instruction type on source accuracies indicated a significant main effect of reference (*F*_(1,15)_ = 5.20, *p* < 0.05, $\eta_{p}^{2}$ = 0.26), and instruction type (*F*_(1,15)_ = 34.42, *p* < 0.001, $\eta_{p}^{2}$ = 0.70), as well as a significant interaction between these two factors (*F*_(1,15)_ = 9.78, *p* < 0.01, $\eta_{p}^{2}$ = 0.40). The subsidiary ANOVAs confirmed that self-referential source accuracies were higher than other-referential source accuracies only for TBR instruction, but there was no significant difference between these two types of source accuracies for TBF instruction. (TBR_SSC − TBR_OSC = 0.07 (0.02) μV; *t*_(15)_ = 3.89, *p* < 0.05; TBF_SSC − TBF_OSC = 0.02 (0.02) μV; *t*_(15)_ = 1.00, *p* = 0.91; Table 1).

**Table 1:** Behavioral results.

	Accuracy	RTs
TBR_S	0.65 (0.10)	1098.61 (124.66)
TBF_S	0.47 (0.09)	1196.29 (133.54)
TBR_O	0.58 (0.11)	1121.90 (123.85)
TBF_O	0.47 (0.11)	1192.53 (142.39)
CR	0.73 (0.13)	1046.95 (133.25)

*Standard deviations in parentheses. Abbreviations: TBR_S, “to be remembered- self”; TBF_S, “to be forgotten-self”; TBR_O, “to be remembered-other”; TBF_O, “to be forgotten-other”; CR, correct rejection.

A similar two-way ANOVA of reference and instruction type on mean RTs of SC trials (source-correct) revealed no reference effect (source memory: *F*_(1,15)_ = 0.61, *p* = 0.446, $\eta_{p}^{2}$ = 0.04), but a significant effect of instruction type (source memory: *F*_(1,15)_ = 26.59, *p* < 0.001, $\eta_{p}^{2}$ = 0.64). The mean RTs for TBR instruction (source-correct: 1110.26 (30.08) ms) were faster than for TBF instruction (source-correct: 1194.41(33.40) ms; Table 1). RTs exceeding ±3 standard deviations from the individual subjects’ mean RT were excluded from analysis.

### ERP Results

According to previous studies of DF (Bailey and Chapman, 2012; Yang et al., 2013), the ERPs were conducted for source memory, in order to explore DF modulation of the self-referential information. The retrieval of source memory was analyzed with its correct-source trials.

**Figure 2:**
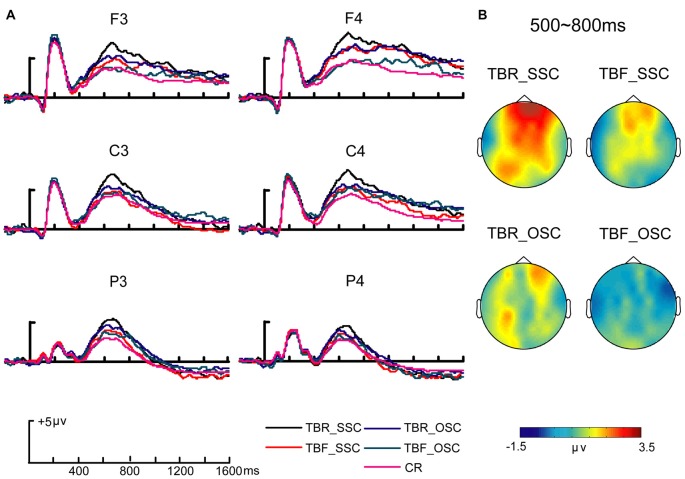
Source-correct event-related potentials (ERPs) of conditions.** (A)** Grand average ERP waveforms of source-correct (SC) trials for “to be remembered- self” (TBR_S), “to be remembered-other” (TBR_O), “to be forgotten-self” (TBF_S) and “to be forgotten-other” (TBF_O) conditions, and correct rejection (CR). **(B)** During late positive complex (LPC; 500–800 ms) time windows of source memory, topographic maps of differential amplitudes showed old-new ERP effects (SC-CR) for “to be remembered-self” (TBR_S), “to be remembered-other” (TBR_O), “to be forgotten-self” (TBF_S) and “to be forgotten-other” (TBF_O) conditions.

The retrieval of recognition was based on two types of processes: familiarity and recollection (Yonelinas, 2002). First, the familiarity processing was demonstrated with the FN400 components (frontal negativity at 300–500 ms). For the 300–500 ms time window of source memory, a repeated-measures ANOVA with factors of condition (TBR_SSC vs. TBF_SSC vs. TBR_OSC vs. TBF_OSC vs. CR) and location (anterior vs. central vs. posterior) indicated significant main effects of condition (*F*_(4,60)_ = 3.78, *p* < 0.05, $\eta_{p}^{2}$ = 0.20), but no significant main effects of location (*F*_(2,30)_ = 0.22, *p* = 0.67, $\eta_{p}^{2}$ = 0.02) or interaction between the two variables (*F*_(8,120)_ = 0.76, *p* = 0.48, $\eta_{p}^{2}$ = 0.05; Figure 2). The planned sample effects showed a significant main effects of condition in posterior region (*F*_(4,60)_ = 3.66, *p* < 0.05, $\eta_{p}^{2}$ = 0.20). The condition effect in posterior region was related to more positive amplitudes for both TBR_SSC and TBF_SSC than CR trials (TBR_SSC − CR = 1.16 (0.29) μV; TBF_SSC − CR = 0.99 (0.23) μV; *t*_(15)_’s > 4.00, *p*s < 0.05), but there was no difference between TBR_SSC and TBF_SSC trials (TBR_SSC − TBF_SSC = 0.17 (0.33) μV; *t*_(15)_ = 0.51, *p* > 0.50). In addition, no difference was observed among TBR_OSC, TBF_OSC and CR trials in posterior region (*t*_(15)_’s < 1.45, *p*s > 0.30). These results suggested significant self-referential effects in the familiarity of source memory without the modulation of DF.

Second, the recollection processing was demonstrated with the LPC effects (parietal potential at 500–800 ms). For the 500–800 ms time window of source memory, a repeated-measures ANOVA with factors of condition (TBR_SSC vs. TBF_SSC vs. TBR_OSC vs. TBF_OSC vs. CR) and location (anterior vs. central vs. posterior) revealed significant main effects of condition (*F*_(4,60)_ = 7.93, *p* < 0.01, $\eta_{p}^{2}$ = 0.35), but no significant main effects of location (*F*_(2,30)_ = 1.04, *p* = 0.33, $\eta_{p}^{2}$ = 0.07) or interaction between the two variables (*F*_(8,120)_ = 1.64, *p* = 0.12, $\eta_{p}^{2}$ = 0.10; Figure 2). The planned sample effects showed a significant main effects of condition in anterior region (*F*_(4,60)_ = 10.92, *p* < 0.001, $\eta_{p}^{2}$ = 0.42) and central region (*F*_(4,60)_ = 5.83, *p* < 0.01, $\eta_{p}^{2}$ = 0.28). The condition effect in anterior region indicated that the TBR_SSC trials evoked higher amplitudes than did the TBF_SSC trials (TBR_SSC − TBF_SSC = 1.66 (0.38), *t*_(15)_ = 4.43, *p* < 0.01), supporting the DF effect. In addition, we found a significant old/new effect for both TBR_SSC and TBR_OSC trials (TBR_SSC − CR = 3.10 (0.37) μV; TBR_OSC − CR = 1.55 (0.39) μV;* t*_(15)_’s > 3.90, *p*s < 0.01), with higher amplitudes for TBR_SSC trials in anterior region (TBR_SSC − TBR_OSC = 1.55 (0.40) μV; *t*_(15)_ = 1.45, *p* < 0.05), suggesting the self-referential effect. The condition effect in anterior region was also related to more positive amplitudes for TBF_SSC than CR trials (TBF_SSC − CR = 1.43 (0.42) μV; *t*_(15)_ = 3.49, *p* < 0.05), with no difference between TBF_OSC and CR trials (TBF_OSC − CR = 0.47 (0.68) μV; *t*_(15)_ = 0.51, *p* = 1.00), thereby supporting that self-referential information alleviated impaired recollection of source memory.

Finally, the late right frontal effects (an amplitude maximum over right frontal scalp at 800–1200 ms) were used to reflect the source monitoring of post-retrieval (Hayama et al., 2008; Cruse and Wilding, 2009). For the 800–1200 ms time window of source memory, a repeated-measures ANOVA with factors of condition (TBR_SSC vs. TBF_SSC vs. TBR_OSC vs. TBF_OSC vs. CR) and location (anterior vs. central vs. posterior) revealed significant main effect of location (*F*_(2,30)_ = 19.03, *p* < 0.001, $\eta_{p}^{2}$ = 0.56), significant main effects of condition (*F*_(4,60)_ = 2.72, *p* = 0.07, $\eta_{p}^{2}$ = 0.15) and interaction between the two variables (*F*_(8,120)_ = 6.22, *p* < 0.001, $\eta_{p}^{2}$ = 0.29; Figure 2). The significant condition effect at the frontal region was observed (*F*_(4,60)_ = 7.93, *p* < 0.001, $\eta_{p}^{2}$ = 0.35), but the condition effect at the central region and parietal region was not significant (central:* F*_(4,60)_ = 1.73, *p* = 0.16, $\eta_{p}^{2}$ = 0.10; parietal:* F*_(4,60)_ = 0.57, *p* = 0.69, $\eta_{p}^{2}$ = 0.04). The condition main effect at the frontal region indicated that the TBR_SSC trials evoked higher amplitudes than the TBR_OSC trials (TBR_SSC − TBR_OSC = 1.35 (0.51) μV; *t*_(15)_ = 2.64, *p* < 0.05), but there was no difference among TBR_SSC, TBF_SSC, TBR_OSC and TBF_OSC trials (*t*_(15)_’s < 1.88, *p* > 0.05). Three types of all condition (TBF_SSC, TBR_OSC, TBF_OSC) evoked no significant old/new effects (TBR_OSC − CR = 2.04 (0.68) μV; TBF_SSC − CR = 2.33 (0.78) μV; TBF_OSC − CR = 1.59 (0.56) μV; *t*_(15)_’s < 2.90, *p*s > 0.08), except for TBR_SSC trials (TBR_SSC − CR = 3.38 (0.42) μV; *t*_(15)_ = 2.73, *p* < 0.001). It suggested that the source monitoring of post-retrieval was enhanced by self-referential information.

## Discussion

Combining a DF paradigm with a source retrieval task, the present study examined whether the DF effects which were alleviated by self-referential information were based on familiarity or recollection. Our behavioral results of the source memory indicated lower accuracies and slower RTs for TBF instruction. These significant DF effects suggested that the retrieval impairment of contextual details due to TBF instruction. The source accuracies of TBF instruction were no difference between self-referential and other-referential information because of floor effects (accuracies were about 0.47). This meant that behavioral results could not suggest that the forgetting alleviation was caused by self-referential information. However, our ERP data, which involved different psychological processes and neural correlates of recognition retrieval, provided evidences for supporting self-referential information that could alleviate DF effects. In line with the behavioral results, neural evidence converged on weakened source memory for TBF instruction, thereby lending support to the notion that DF effects impaired source memory. With enhanced FN400 and LPC for self-referential information, the self-referential effects were demonstrated to be reflected in familiarity and recollection. Furthermore, the LPC components of self-referential memory in TBF condition suggested that the DF effects which were alleviated by self-referential information were based on recollection processes (rather than familiarity).

The retrieval of self-referential information was based on two types of recognition processes: familiarity and recollection (Yonelinas, 2002). The FN400 component which indexed familiarity processes was sensitive to memory strength (Curran, 2000; Diana et al., 2007; Rugg and Curran, 2007; Woroch and Gonsalves, 2014). Also, FN400 components had contribution to familiarity of source memory (Mollison and Curran, 2012). Our FN400 effects of source memory suggested that self-referential effects promoted familiarity processes. These effects were not modulated by TBF instruction, reflecting that the memory strength of source memory was affected by self-referential processing, rather than TBF instruction. Generally, the previous ERP studies endorsed that familiarity processes could not support self-referential effects (Magno and Allan, 2007). Surprisingly, the familiarity of source memory was improved by self-referential information with more self-semantic knowledge in the present study, because FN400 were demonstrated to be enhanced by increased semantic information (Hou et al., 2013).

Furthermore, the amplitudes of LPC old/new effects in TBR condition were enhanced for the self-referential source memory, indicating that self-referential processing improved the recollection of contextual details. Consistent with previous research, our findings agreed that self-reference process could promote recollection of source details (Dulas et al., 2011; Serbun et al., 2011). These significant self-referential effects of source memory could be attributed to autobiographical retrieval, especially with nouns (Klein and Kihlstrom, 1986; Klein, 2012). The autobiographical retrieval consists of specific items of personal information that are closely related to unique autobiographical events which refer to the individual in relation to a specific episodic context (Matuszewski et al., 2009). Our source judgment task asked subjects to judge whether the item was self-referential or other-referential. In order to make more accurate decision of this task, subjects trended to recollect the episodic context of the study phase. It was easier to recollect that self-referential nouns were related to autobiographical experience of subjects. For example, “my table” was easy to elicit autobiographical experience of my own table in real life; while “his sofa” was hard to construct episodic context, because the sofa of Chinese Prime Minister was seldom presented (our experiment instructed participants to consider “his-noun” pairs as the things related to Chinese Prime Minister). This autobiographical retrieval of source memory provided plenty of episodic context for self-referential source judgment. Thus, source memory tasks of self-referential information were more likely to elicit autobiographical retrieval, which could enhance self-referential retrieval advantages.

Our ERP evidence supported that autobiographical retrieval happened for self-referential information. On one hand, self-referential effects in the present study were evoked by nouns which were failing to obtain self-referential effects without requiring autobiographical retrieval (Klein and Kihlstrom, 1986). On the other hand, our result revealed an activated LPC component in the frontal region which was associated with autobiographical retrieval, though it has been reported that the LPC component was maximal over the parietal scalp (Curran, 2000; Diana et al., 2007; Rugg and Curran, 2007). The frontal positivity of the LPC component during recollection was sensitive to episodic content of retrieval and autobiographical retrieval, supporting our LPC results at frontal (Peters and Daum, 2009; Mitchell and Johnson, 2009). The prefrontal cortex was often proved to be related to self-referential processing, particularly autobiographical retrieval (Martinelli et al., 2013). The fMRI studies showed that the amount of activity in FPC (prefrontal cortex) correlated with autobiographical retrieval and recollection of self-referential items (Amodio and Frith, 2006; Vinogradov et al., 2006; Turner et al., 2008; Wylie et al., 2008; Oddo et al., 2010; Leshikar and Duarte, 2012; Morel et al., 2014). Additionally, the enhanced late right frontal wave of self-referential information could be explained by the observation that the processing of post-retrieval reorganization and source monitoring might be facilitated by autobiographical retrieval.

However, the behavioral and neural evidence converged on the notion that the DF effects which was involved with recollection was alleviated by self-referential information. Different with our FN400 effects of source memory which was not influenced by TBF instruction, the decreased LPC effects of source memory indicated that retrieval inhibition of recollection which was evoked by TBF instruction. Substantial evidence of the absence of the LPC effects has been accrued for inhibition retrieval in item method (Ullsperger et al., 2000). In contrast to the absence of LPC old/new effects for other-referential trials, the self-referential trials elicited the significant LPC old/new effects for TBF instruction. The evidence aligned with recent reports in the literature and agreed that self-referential information could alleviate DF effects (Sahakyan and Delaney, 2005; Racsmány and Conway, 2006; Bastin et al., 2012; Yang et al., 2013). As LPC old/new effects was associated with recollection, our ERP evidences supported that self-referential information overcame retrieval inhibition of DF, which was based on recollection processes. This alleviation of the recollection inhibition was also attributed to autobiographical retrieval caused by self-referential information. With recollecting episodic details in real life, this autobiographical retrieval of source memory provided plenty of retrieval-cues for reactivating the memory representation of self-referential items (Uncapher and Rugg, 2009; Turk et al., 2013). Thus, it suggested self-referential information could alleviate retrieval inhibition which was associated with recollection.

Therefore, our neural findings and behavioral results investigated the influence of DF on source memory for self-referential information, demonstrating that the self-referential information alleviated DF effects. The self-referential processing enhanced behavioral accuracies and ERP amplitudes of FN400 and LPC effects, suggesting that self-referential processing improved both familiarity and recollection. Our neural findings were consistent with our behavioral results suggesting that the retrieval inhibition of DF effects which were associated with recollection processes were alleviated by the self-referential information due to autobiographical retrieval.

### Limitations

The current study poses some notable limitations. First, the sample size in the current study is small, due to the limited time and finance. To acquire more robust conclusion in future studies, large simple size of participants should be used in the next research. Second, ERP data of retrieval phase in our research was reported, but the ERP data of encoding phase was not recorded. ERP data of encoding phase should be analyzed to compare with retrieval phase so that we can obtain the association between encoding phase and retrieval phase to explain the alleviative DF caused by self-referential processing. Third, we acknowledged that Bonferroni-corrected *post hoc* pair-wise comparisons could not be valid enough to correct for the number of independent ANOVA analyses and our results should be verified with caution in further research. Finally, the number of stimuli per category is not large and might lead to the low signal to noise ratio. More trials of each category should be designed to obtain more precise signal of ERP data.

## Author Contributions

CG and YWu: supervised the project and designed the study. YWang revised the draft of manuscript. XM analyzed the data and wrote the main manuscript text. All authors reviewed the manuscript.

## Conflict of Interest Statement

The authors declare that the research was conducted in the absence of any commercial or financial relationships that could be construed as a potential conflict of interest.

## References

[B100] Addante R. J. Ranganath C. Yonelinas A. P. (2012). Examining ERP correlates of recognition memory: evidence of accurate source recognition without recollection. Neuroimage 62, 439–450. 10.1016/j.neuroimage.2012.04.031 22548808 PMC3381051

[B1] Amodio D. M. Frith C. D. (2006). Meeting of minds: the medial frontal cortex and social cognition. Nat. Rev. Neurosci. 7, 268–277. 10.1038/nrn1884 16552413

[B2] Bailey K. Chapman P. (2012). When can we choose to forget? An ERP study into item-method directed forgetting of emotional words. Brain Cogn. 78, 133–147. 10.1016/j.bandc.2011.11.004 22240487

[B3] Basden D. R. Gargano G. J. (1993). Directed forgetting in implicit and explicit memory tests: a comparison of methods. J. Exp. Psychol. Learn. Mem. Cogn. 19, 603–616. 10.1037//0278-7393.19.3.603

[B5] Bastin C. Feyers D. Majerus S. Balteau E. Degueldre C. Luxen A.. (2012). The neural substrates of memory suppression: a fMRI exploration of directed forgetting. PLoS One 7:e29905. 10.1371/journal.pone.0029905 22238671 PMC3253105

[B4] Bjork R. A. (1989). “Retrieval inhibition as an adaptive mechanism in human memory,” in Varieties of Memory and Consciousness: Essays in Honour of Endel Tulving, eds Roediger H. L. Craik F. I. M. (Hillsdale, NJ: Erlbaum), 309–330.

[B6] Cruse D. Wilding E. L. (2009). Prefrontal cortex contributions to episodic retrieval monitoring and evaluation. Neuropsychologia 47, 2779–2789. 10.1016/j.neuropsychologia.2009.06.003 19523968

[B7] Cunningham S. J. Brady-Van den Bos M. Turk D. J. (2011). Exploring the effects of ownership and choice on self-memory biases. Memory 19, 449–461. 10.1080/09658211.2011.584388 21864211

[B8] Curran T. (2000). Brain potentials of recollection and familiarity. Mem. Cognit. 28, 923–938. 10.3758/bf03209340 11105518

[B9] Diana R. A. Yonelinas A. P. Ranganath C. (2007). Imaging recollection and familiarity in the medial temporal lobe: a three-component model. Trends Cogn. Sci. 11, 379–386. 10.1016/j.tics.2007.08.001 17707683

[B10] Dulas M. R. Newsome R. N. Duarte A. (2011). The effects of aging on ERP correlates of source memory retrieval for self-referential information. Brain Res. 1377, 84–100. 10.1016/j.brainres.2010.12.087 21215731

[B11] Gratton G. Coles M. G. Donchin E. (1983). A new method for off-line removal of ocular artifact. Electroencephalogr. Clin. Neurophysiol. 55, 468–484. 10.1016/0013-4694(83)90135-9 6187540

[B12] Guo C. Duan L. Li W. Paller K. A. (2006). Distinguishing source memory and item memory: brain potentials at encoding and retrieval. Brain Res. 1118, 142–154. 10.1016/j.brainres.2006.08.034 16978588

[B13] Hayama H. R. Johnson J. D. Rugg M. D. (2008). The relationship between the right frontal old/new ERP effect and post-retrieval monitoring: specific or nonspecific? Neuropsychologia 46, 1211–1223. 10.1016/j.neuropsychologia.2007.11.021 18234241 PMC2441597

[B14] Hou M. Safron A. Paller K. A. Guo C. (2013). Neural correlates of familiarity and conceptual fluency in a recognition test with ancient pictographic characters. Brain Res. 1518, 48–60. 10.1016/j.brainres.2013.04.041 23632379

[B16] Klein S. B. (2012). Self, memory, and the self-reference effect an examination of conceptual and methodological issues. Pers. Soc. Psychol. Rev. 16, 283–300. 10.1177/1088868311434214 22291045

[B17] Klein S. B. Kihlstrom J. F. (1986). Elaboration, organization, and the self-reference effect in memory. J. Exp. Psychol. Gen. 115, 26–38. 10.1037//0096-3445.115.1.26 2937872

[B18] Leshikar E. D. Duarte A. (2012). Medial prefrontal cortex supports source memory accuracy for self-referenced items. Soc. Neurosci. 7, 126–145. 10.1080/17470919.2011.585242 21936739 PMC3701388

[B19] Levy B. J. Anderson M. C. (2008). Individual differences in the suppression of unwanted memories: the executive deficit hypothesis. Acta Psychol. (Amst) 127, 623–635. 10.1016/j.actpsy.2007.12.004 18242571

[B20] Liu Y. Liang N. Wang D. Zhang S. Yang T. Jie C. (1990). Mordern Chinese Word Frequency Dictionary for Commonly Used Words: The Sequencer Part. Beijing: China Astronautic Publishing House.

[B21] MacLeod C. M. (1998). “Directed forgetting,” in Intentional Forgetting: Interdisciplinary Approaches, eds Golding J. M. MacLeod C. M. (Mahwah, NJ: Erlbaum), 1–58.

[B22] Magno E. Allan K. (2007). Self-reference during explicit memory retrieval: an event-related potential analysis. Psychol. Sci. 18, 672–677. 10.1111/j.1467-9280.2007.01957.x 17680935

[B23] Martinelli P. Sperduti M. Piolino P. (2013). Neural substrates of the self-memory system: new insights from a meta-analysis. Hum. Brain Mapp. 34, 1515–1529. 10.1002/hbm.22008 22359397 PMC6870171

[B24] Matuszewski V. Piolino P. Belliard S. de la Sayette L. Laisney M. Lalevée C.. (2009). Patterns of autobiographical memory impairment according to disease severity in semantic dementia. Cortex 45, 456–472. 10.1016/j.cortex.2007.11.006 19231476

[B25] Mecklinger A. Parra M. Waldhauser G. T. (2009). ERP correlates of intentional forgetting. Brain Res. 1255, 132–147. 10.1016/j.brainres.2008.11.073 19103178

[B26] Mitchell K. J. Johnson M. K. (2009). Source monitoring 15 years later: what have we learned from fMRI about the neural mechanisms of source memory? Psychol. Bull. 135, 638–677. 10.1037/a0015849 19586165 PMC2859897

[B27] Mollison M. V. Curran T. (2012). Familiarity in source memory. Neuropsychologia 50, 2546–2565. 10.1016/j.neuropsychologia.2012.06.027 22789677 PMC3432179

[B28] Morel N. Villain N. Rauchs G. Gaubert M. Piolino P. Landeau B.. (2014). Brain activity and functional coupling changes associated with self-reference effect during both encoding and retrieval. PLoS One 9:e90488. 10.1371/journal.pone.0090488 24608131 PMC3946483

[B29] Oddo S. Lux S. Weiss P. H. Schwab A. Welzer H. Markowitsch H. J.. (2010). Specific role of medial prefrontal cortex in retrieving recent autobiographical memories: an fMRI study of young female subjects. Cortex 46, 29–39. 10.1016/j.cortex.2008.07.003 19084220

[B15] Peters J. Daum I. (2009). Frontal but not parietal positivity during source recollection is sensitive to episodic content. Neurosci. Lett. 454, 182–186. 10.1016/j.neulet.2009.03.019 19429080

[B30] Racsmány M. Conway M. A. (2006). Episodic inhibition. J. Exp. Psychol. Learn. Mem. Cogn. 32, 44–57. 10.1037/0278-7393.32.1.44 16478339

[B31] Racsmány M. Conway M. A. Garab E. A. Nagymáté G. (2008). Memory awareness following episodic inhibition. Q. J. Exp. Psychol. (Hove) 61, 525–534. 10.1080/17470210701728750 18300184

[B32] Rogers T. B. Kuiper N. A. Kirker W. S. (1977). Self-reference and the encoding of personal information. J. Pers. Soc. Psychol. 35, 677–688. 10.1037//0022-3514.35.9.677 909043

[B33] Rugg M. D. Curran T. (2007). Event-related potentials and recognition memory. Trends Cogn. Sci. 11, 251–257. 10.1016/j.tics.2007.04.004 17481940

[B34] Sahakyan L. Delaney P. F. (2005). Directed forgetting in incidental learning and recognition testing: support for a two-factor account. J. Exp. Psychol. Learn. Mem. Cogn. 31, 789–801. 10.1037/0278-7393.31.4.789 16060780

[B35] Serbun S. J. Shih J. Y. Gutchess A. H. (2011). Memory for details with self-referencing. Memory 19, 1004–1014. 10.1080/09658211.2011.626429 22092106 PMC3226761

[B36] Symons C. S. Johnson B. T. (1997). The self-reference effect in memory: a meta-analysis. Psychol. Bull. 121, 371–394. 10.1037//0033-2909.121.3.371 9136641

[B37] Turk D. J. Brady-van den Bos M. Collard P. Gillespie-Smith K. Conway M. A. Cunningham S. J. (2013). Divided attention selectively impairs memory for self-relevant information. Mem. Cognit. 41, 503–510. 10.3758/s13421-012-0279-0 23263878

[B38] Turner M. S. Simons J. S. Gilbert S. J. Frith C. D. Burgess P. W. (2008). Distinct roles for lateral and medial rostral prefrontal cortex in source monitoring of perceived and imagined events. Neuropsychologia 46, 1442–1453. 10.1016/j.neuropsychologia.2007.12.029 18294660 PMC2697314

[B39] Ullsperger M. Mecklinger A. Müller U. (2000). An electrophysiological test of directed forgetting: the role of retrieval inhibition. J. Cogn. Neurosci. 12, 924–940. 10.1162/08989290051137477 11177414

[B40] Uncapher M. R. Rugg M. D. (2009). Selecting for memory? The influence of selective attention on the mnemonic binding of contextual information. J. Neurosci. 29, 8270–8279. 10.1523/JNEUROSCI.1043-09.2009 19553466 PMC2730727

[B41] van den Bos M. Cunningham S. J. Conway M. A. Turk D. J. (2010). Mine to remember: the impact of ownership on recollective experience. Q. J. Exp. Psychol. (Hove) 63, 1065–1071. 10.1080/17470211003770938 20401814

[B42] Van Hooff J. C. Whitaker T. A. Ford R. M. (2009). Directed forgetting in direct and indirect tests of memory: seeking evidence of retrieval inhibition using electrophysiological measures. Brain Cogn. 71, 153–164. 10.1016/j.bandc.2009.05.001 19556048

[B43] Vinogradov S. Luks T. L. Simpson G. V. Schulman B. J. Glenn S. Wong A. E. (2006). Brain activation patterns during memory of cognitive agency. Neuroimage 31, 896–905. 10.1016/j.neuroimage.2005.12.058 16516497

[B44] Voss J. L. Paller K. A. (2008). 3.05 neural substrates of remembering–electroencephalographic studies. Learn. Mem. 3, 79–97. 10.1016/b978-012370509-9.00106-6

[B45] Woodward A. E. Jr. Bjork R. A. Jongeward R. H. Jr. (1973). Recall and recognition as a function of primary rehearsal. J. Verbal Learn. Verbal Behav. 12, 608–617. 10.1016/s0022-5371(73)80040-4

[B46] Woroch B. Gonsalves B. D. (2014). Event-related potential correlates of item and source memory strength. Brain Res. 1317, 180–191. 10.1016/j.brainres.2009.12.074 20051237 PMC2897745

[B47] Wylie G. R. Foxe J. J. Taylor T. L. (2008). Forgetting as an active process: an fMRI investigation of item-method-directed forgetting. Cereb. Cortex 18, 670–682. 10.1093/cercor/bhm101 17617657

[B48] Xiao X. Zhao D. Zhang Q. Guo C. Y. (2012). Retrieval of concrete words involves more contextual information than abstract words: multiple components for the concreteness effect. Brain Lang. 120, 251–258. 10.1016/j.bandl.2011.09.006 22041121

[B49] Yang W. Liu P. Cui Q. Wei D. Li W. Qiu J.. (2013). Directed forgetting of negative self-referential information is difficult: an fMRI study. PLoS One 8:e75190. 10.1371/journal.pone.0075190 24124475 PMC3790724

[B50] Yonelinas A. P. (2002). The nature of recollection and familiarity: a review of 30 years of research. J. Mem. Lang. 46, 441–517. 10.1006/jmla.2002.2864

